# Needle insufflation into the liver as a cause of massive gas embolus and CVA

**DOI:** 10.1093/jscr/rjab448

**Published:** 2021-10-27

**Authors:** Pamela G McIntosh, Chris G Andrew

**Affiliations:** Department of Surgery, University of Manitoba, Winnipeg, MB, Canada; Department of Surgery, University of Manitoba, Winnipeg, MB, Canada

## Abstract

Laparoscopy is being applied more frequently and in broader applications. Complications of this technique are infrequent, and rare among them are gas emboli due to insufflation. This paper describes a 65-year-old obese female presenting for elective laparoscopic cholecystectomy who suffered a cerebral vascular accident after Veress needle insertion into undiagnosed severe fatty liver led to a massive gas embolus. Our patient experienced immediate cardiac compromise and acute monoparesis. Intra-operative transesophageal echocardiogram revealed copious air in the right atria and ventricle. A needle track within the liver was visible on a post-operative computerized tomography scan. The patient made a full recovery, but this acts as a reminder to be vigilant for potential complications of laparoscopy and highlights challenges of laparoscopic entry in the severely obese.

## INTRODUCTION

Complications related to laparoscopy have been estimated between 0.2 and 10.3%, with entry injuries 0.3–1.0% [1]. Gas embolus (also known as ‘carbon dioxide [CO_2_] embolus’) is a rare complication, resulting from insufflation to create the pneumoperitoneum [2–4]. There are case reports of gas embolus in laparoscopic surgery, which typically occur partway through the case due vascular injury [5–10]. Cases of gas embolus with insufflation have also been reported in the setting of an angiomyolipoma with suspected abnormal vasculature [11] and in a case of abnormal superior vena cava anatomy [12]. We present a unique case of carbon dioxide embolus occurring upon initial access to the abdomen with insufflation needle misplacement into the liver, in the setting of severe obesity. This resulted in creation of a massive gas embolus, cardiorespiratory compromise and cerebral vascular accident (CVA), leading to temporary monoparesis.

## CASE REPORT

A 65-year-old Caucasian female (height, 156 cm; weight, 123 kg; body mass index [BMI], 50.5) presented for elective laparoscopic cholecystectomy for recurrent biliary colic. There were almost 2 years between her initial ambulatory evaluation and the index operation, prolonged by the COVID-19 pandemic.

Apart from severe obesity, her medical history included obstructive sleep apnea (with home continuous positive airway pressure therapy, CPAP), type 2 diabetes, hypertension, dyslipidemia and reflux. She had no prior history of abdominal surgeries, smoking or alcohol. Pre-operative stress test, almost 4 years prior, was normal. Family history was non-contributory.

On the day of the operation, pre-operative vital signs were as follows: blood pressure, 145/82; heart rate, 86; SpO_2_, 96% on room air. The patient received general anesthetic per standard protocol. In preparation for Veress needle insufflation, a small skin incision was made at Palmer’s point, below the costal margin in the left upper quadrant. The needle was inserted deeply at 90°, with no flash of gas or blood. Saline drop did drip through. CO_2_ insufflation was initiated at 2 L/min, and pressures were noted to be higher than expected. Almost immediately, SpO_2_ dropped from 97% to 67%. Insufflation tubing was detached, and the Veress needle was removed. Oxygen saturations remained low, mean arterial pressure dropped to <50, end title CO_2_ (ETCO_2_) dropped to 8. Cardiac monitoring showed sinus tachycardia with ST segment elevation. Epinephrine was required for cardiac support.

Initial differential diagnosis included possible Veress misplacement into the thoracic cavity, resulting in pneumothorax. However, immediate decompression of the left chest cavity with a 14-gauge catheter, followed by finger thoracostomy and chest tube placement, did not demonstrate any gas or blood. Cardiac anesthesia performed transesophageal echocardiogram (TEE), and copious gas was seen in the right atrium and ventricle, associated right ventricular dysfunction, and estimated low cardiac output. A right intrajugular catheter was inserted, but unable to aspirate any gas. In the subsequent 20 min, with ongoing bag-ventilation, saturations improved to 97%. Epinephrine was weaned and ST elevation resolved. Repeat TEE showed dissolution of gas in cardiac chambers, normalization of biventricular function, no pericardial effusion and no clot.

**Figure 1:**
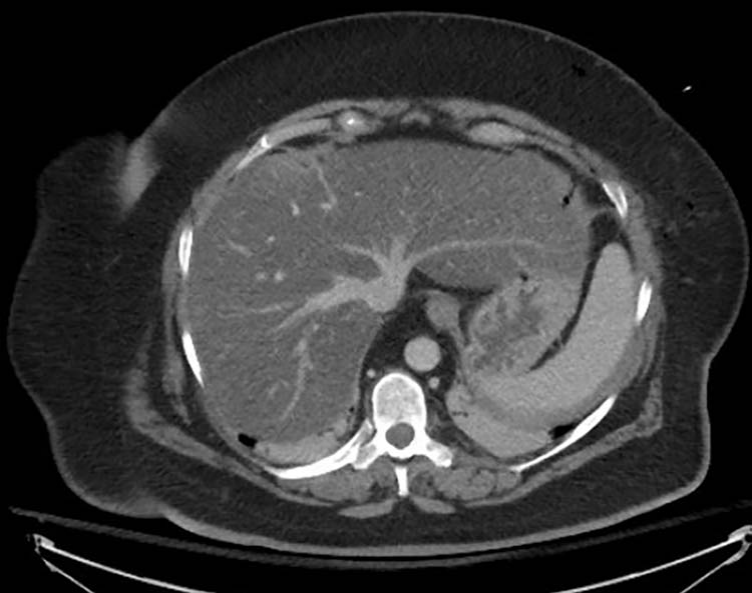
CT thorax and abdomen, infused, immediately post-operative noting severe fatty infiltration within the liver and Veress needle track.

The planned cholecystectomy was not re-attempted, and the patient remained intubated. She underwent emergent infused CT chest and abdomen to rule out other injuries (Fig. 1). It showed severe fatty infiltration of the liver, with tracking of gas into the liver parenchyma. No other abnormalities were noted. It was evident that the needle had entered the liver, leading to a gas embolus. Uninfused CT brain and CT angiography later that day were unremarkable with no evidence of infarction and no abnormalities noted within cranial circulation.

The patient was extubated on post-operative day (POD) 1. On awakening, she had left arm paresis. She was unable to flex her left shoulder or elbow but did have a weak grip strength. Right arm, and bilateral leg had preserved motor function. By the end of the day POD 1, she was able to hold both arms overhead, but still had left sided weakness and poor coordination. By POD 2, she was noted to have full range of motion, still with slight weakness on left. MRI Brain on POD 3 showed ‘subtle diffusion restriction of the cortex of the right precentral gyrus consistent with right MCA acute non-hemorrhagic infarction.’ A formal transthoracic echocardiogram confirmed a patent foramen ovale.

Full arm strength returned by POD 3. The patient continued to improve and was discharged on POD 5. Written consent was completed by the patient for publication of her case.

## DISCUSSION

Clinically significant gas embolus from needle insufflation into a vascular organ is a rarely reported event. Gas emboli present as acute onset hypoxia, drop in ETCO_2_, hemodynamic compromise (hypotension) and arrythmias [3, 4]. Differential diagnosis includes anaphylaxis, coronary ischemia, pneumothorax or hemorrhage secondary to vascular injury [3, 4]. TEE is the most sensitive method of detection [3]. Management includes positional change to Trendelenburg and left lateral decubitus, cease insufflation, attempt aspiration of the gas via central access, and cardiopulmonary support at indicated [3, 4]. In this case, the patent foramen ovale would have allowed gas to move in a right-to-left shunt then through arterial circulation to the brain, leading to the right MCA infarction.

In this case, the patient’s morbid obesity and severe fatty liver was likely a major contributor, increasing the likelihood of needle perforation even though Veress insertion at Palmer’s point is considered one of the safest entry methods in obese patients [13]. Obese patients present unique challenges of access, due to typically thicker abdominal walls and distorted anatomy from a large pannus, reducing the utility of the umbilicus as a reliable landmark [13, 14]. Specific recommendations lack supportive data. Even the recent Cochrane review comparing safety of various laparoscopic entry techniques noted most randomized control trials excluded patients with higher BMI, thus limiting generalizability of results [15].

In conclusion, we presented a case of a routine laparoscopic cholecystectomy which ended in cardiac compromise and temporary monoplegia due to gas embolus created on insufflation. This acts as a reminder to be vigilant for potential complications of laparoscopy and highlights challenges of laparoscopic entry in the morbidly obese, a dilemma which will be more frequently encountered as the prevalence of obesity increases.
